# Quantitative Analysis of Serum Procollagen Type I C-Terminal Propeptide by Immunoassay on Microchip

**DOI:** 10.1371/journal.pone.0018807

**Published:** 2011-04-13

**Authors:** Shouki Yatsushiro, Rie Akamine, Shohei Yamamura, Mami Hino, Kazuaki Kajimoto, Kaori Abe, Hiroko Abe, Jun-ichi Kido, Masato Tanaka, Yasuo Shinohara, Yoshinobu Baba, Toshihiko Ooie, Masatoshi Kataoka

**Affiliations:** 1 Health Research Institute, National Institute of Advanced Industrial Science and Technology (AIST), Japan; 2 Division of Medico-Dental Dynamics and Reconstruction, Department of Periodontology and Endodontology, Oral and Maxillofacial Dentistry, Institute of Health Biosciences, University of Tokushima, Tokushima, Japan; 3 Faculty of Pharmaceutical Sciences, University of Tokushima, Tokushima, Japan; 4 Institute for Genome Research, University of Tokushima, Tokushima, Japan; 5 Department of Applied Chemistry, Graduate School of Engineering Nagoya University, Nagoya, Japan; University of California, Merced, United States of America

## Abstract

**Background:**

Sandwich enzyme-linked immunosorbent assay (ELISA) is one of the most frequently employed assays for clinical diagnosis, since this enables the investigator to identify specific protein biomarkers. However, the conventional assay using a 96-well microtitration plate is time- and sample-consuming, and therefore is not suitable for rapid diagnosis. To overcome these drawbacks, we performed a sandwich ELISA on a microchip.

**Methods and Findings:**

The microchip was made of cyclic olefin copolymer with straight microchannels that were 300 µm wide and 100 µm deep. For the construction of a sandwich ELISA for procollagen type I C-peptide (PICP), a biomarker for bone formation, we used a piezoelectric inkjet printing system for the deposition and fixation of the 1st anti-PICP antibody on the surface of the microchannel. After the infusion of the mixture of 2.0 µl of peroxidase-labeled 2nd anti-PICP antibody and 0.4 µl of sample to the microchannel and a 30-min incubation, the substrate for peroxidase was infused into the microchannel; and the luminescence intensity of each spot of 1st antibody was measured by CCD camera. A linear relationship was observed between PICP concentration and luminescence intensity over the range of 0 to 600 ng/ml (r^2^ = 0.991), and the detection limit was 4.7 ng/ml. Blood PICP concentrations of 6 subjects estimated from microchip were compared with results obtained by the conventional method. Good correlation was observed between methods according to simple linear regression analysis (R^2^ = 0.9914). The within-day and between-days reproducibilities were 3.2–7.4 and 4.4–6.8%, respectively. This assay reduced the time for the antigen-antibody reaction to 1/6, and the consumption of samples and reagents to 1/50 compared with the conventional method.

**Conclusion:**

This assay enabled us to determine serum PICP with accuracy, high sensitivity, time saving ability, and low consumption of sample and reagents, and thus will be applicable to clinic diagnosis.

## Introduction

Many plasma proteins that have originated from various tissues or blood cells as a result of secretion or leakage can be used as biomarkers, and many studies have demonstrated that plasma protein levels reflect human physiological or pathological states and can thus be used for disease diagnosis and prognosis [1]–[3]. The immunoassay is often employed in clinical and experimental laboratories, since this technique enables the identification of specific protein biomarkers; and small amounts of target molecules present in complex biological samples such as blood can be specifically detected by its use. The double antibody sandwich technique, the so-called sandwich enzyme-linked immunosorbant assay (ELISA), is frequently used for antigen measurement [4], [5]. This assay is specifically useful for detecting a specific antigen when only a small amount of it is available and purified antigen is unavailable. In the conventional sandwich ELISA system, disposable 96-well microtitration plates are frequently employed to detect biomarkers in blood. Typically 50-µl aliquots of capture antibody (1st antibody) and enzyme conjugated antibody (2nd antibody) to detect antigen, and 50 µl aliquots of antigen solution are employed in the reaction wells in 96-well microtitration plates [6]. Furthermore, this method is time-consuming, for the capture of antigen by 1st antibody and the determination of the antigen concentration by the 2nd antibody each requires 2 hr or more. So, the conventional sandwich ELISA is not suitable for rapid diagnosis.

The analysis of biomarkers at a location near the patient, which analysis is called point-of care testing (POCT), is a continuously expanding trend in the practice of laboratory diagnosis [7], and it is preferred when test results are needed more rapidly than is feasible by using conventional testing procedures [8]–[11]. Several types of microchips have been developed for chemical and biological analyses [12]–[15], and several types of immunoassay on a microchip have been applied practically in POCT [16]–[24]. Such analysis using microchips has many advantages such as high efficiency, time-saving ability, easy operation, low consumption of samples and reagents, and easy integration and automation. Antibody adsorbed to beads-packed in the microchannels have often been employed for sandwich ELISA [19], [21]–[24]. For these immunoassays, elaborate microscopic dam-like structures (a few score or so of µm scale gaps) in the microchannel are needed to stop these microbeads in suspension from flowing throughout the microchannel. Recently, inkjet printing systems to deposit and immobilize an antibody on a nylon membrane were used for immunoassay application [25]. Inkjet printing allows for the straightforward deposition of biological materials on the assay surface. For the development of sandwich ELISA assays on the microchip, we employed piezoelectric inkjet printing for deposition and fixation of the 1st antibody on the microchannel surface.

In the present study, the model analyte used was the carboxyterminal propeptide of type I procollagen (PICP). Determination of the PICP level is a means of estimating the rate of type I collagen synthesis in the body, and the serum concentration of PICP is known to correlate with the rate of bone formation [26]. Therefore, the serum concentration of PICP can be used as a biomarker for osteoporosis, and osteoblastic bone metastases in prostatic cancer. A piezoelectric inkjet printing system capable of creating sub-millimeter patterns of aqueous reagent with precise placement for biosensor applications was recently developed [27]. In the present study anti-PICP 1st antibody was deposited and fixed on the microchannel surface by using this inkjet printing technique, and a sandwich ELISA assay using 0.4 µl of serum for PICP determination was performed in the microchannel. We examined the feasibility of determining serum concentration of PICP with this system. The accuracy of determining the serum concentration of PICP in a microchannel was comparable to that obtained by the conventional sandwich ELISA using the 96-well microtitration plates. Furthermore, the time required for the immunoassay was drastically shortened. We demonstrated the possible application of microchips for monitoring serum PICP levels and its potential for POCT.

## Methods

### Principle

**Figure 1:**
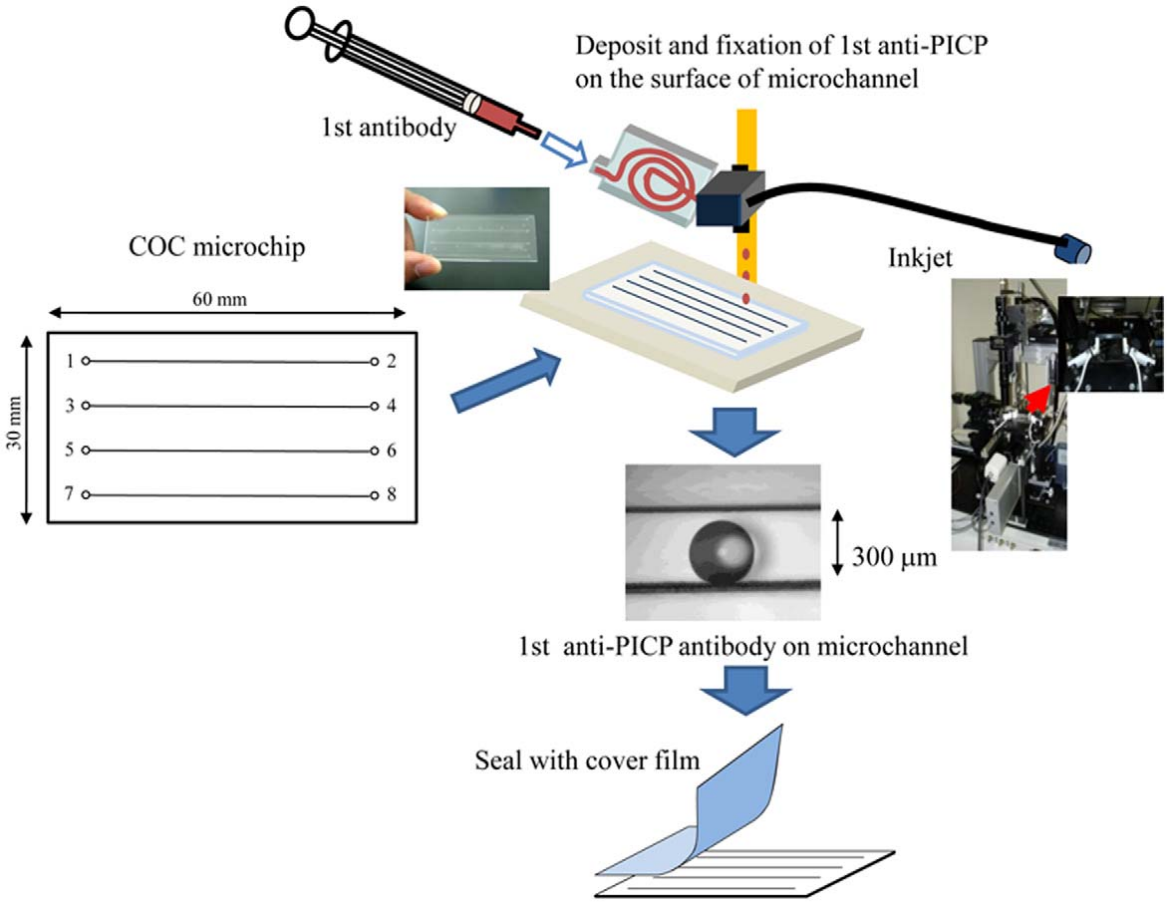
Schematic process for detection of PICP on a microchip with deposition and fixation of 1st antibody by piezoelectric ink printing. After fixation of antibody on the surface of the microchannel and sealing with a cover film, blocking solution was infused into each microchannel from the reservoir (wells 1, 3, 5, and 7) by use of a pipetman.

Schematic illustrations of sandwich ELISA assay on microchip for the determination of serum PICP are shown in Fig. 1. The microchip was made of cyclic olefin copolymer (COC) and was 30 mm long and 60 mm wide. The channels in the microchip were straight, 300 µm wide, and 100 µm deep. The COC microchip was fabricated by injection molding with a nickel mold, and the microchip surface was treated with polymer solution containing p-nitrophenyl ester which binds to amino groups in proteins for 1st antibody fixation on microchannel surface (BS-X2321, SUMITOMO BAKELITE Co., Ltd, Tokyo, Japan). This microchip was used in this immunoassay for the detection of serum PICP. Four samples could be analyzed simultaneously on the microchip. A Pulseinjector (Cluster Technology Co., Ltd., Osaka, Japan), which is the piezoelectric inkjet printing system, was employed for the deposition and fixation of the 1st antibody on the microchannel. The derive waveform C was employed to provide the volume of droplet, and the droplets were ejected at a frequency of 20 Hz and a jetting voltage of 14 V. The volume of a single discharged droplet of 1st antibody was 65 pl, and 100 droplets were discharged onto the surface of the microchannel to fix the maximum volume of the 1st antibody as shown in Fig. 1. After the deposition and fixation of the 1st anti-PICP antibody on the surface of the microchannel, the microchip surface was sealed by a cover film made of polymethylmethacrylate and having a 33-µm thickness and 20-µm adhesion layer (TOYO INK MFG. CO., LTD. Tokyo, Japan). All samples and reagents for the sandwich ELISA assay were infused from reservoirs with a diameter of 1.0 mm (wells 1, 3, 5, and 7) into the microchannels by a pipette. The reservoirs on the other side were used as waste reservoir (wells 2, 4, 6, and 8).

### Reagents and analytical procedure

A procollagen type I C-peptide (PIP) EIA Kit (precoated) was purchased from TAKARA BIO INC. (Shiga, Japan) and employed for the determination of the serum concentration of PICP with a microtitration plate as the conventional method according to the supplied instructions. Briefly, 100 µl of peroxidase-labeled anti-PICP 2nd antibody and 20 µl of sample were simultaneously added to each well of a 96-well microtitration plate that had been precoated with anti-PICP 1st antibody, and the plate was then incubated for 3 hr at room temperature. To make a calibration curve we diluted PICP with sample diluent to 600, 300, and 150 ng/ml; and these were used as samples. For the determination of the serum concentration of PICP, serum was diluted 5 times with sample diluent. After the plate had been washed with phosphate-buffered saline, 100 µl of substrate solution for peroxidase was added to the wells, and the plate was then incubated for 15 min at room temperature. After the addition of the stop solution to the wells, the absorbance of the solution was measured at 450 nm by using a microplate reader (Tecan Austria GmbH, Salzburg, Austria). The serum concentration of PICP was calculated from the standard curve and expressed as ng/ml.

### Analysis of PICP on the microchip

For the sandwich ELISA assay on the microchip, we used the same monoclonal anti-PICP antibodies that were used in the Procollagen type I C-peptide (PIP) EIA Kit for 96-well of microtitration plates and substrates. For the deposition and fixation of 0.1 mg/ml 1st antibody (Clone PC8-7, TAKARA) in spotting buffer on the surface of the microchannel, a piezoelectric inkjet printing system (Cluster Technology Co., Ltd. Osaka, Japan) was employed. A jetting voltage of 14 V for antibody “inks” was used in this study. The volume of the drop ejected from the inkjet nozzle was 65 pl, and 100 drops were spotted onto the microchannel surface, as shown in Figure 1. The microchip was then incubated for 4 hr at room temperature. Spots were round with a 250-µm diameter. After sealing of the microchip surface with the cover film, blocking solution was infused into each microchannel; and the microchip was left undisturbed for 1 hr at room temperature. Washing of the microchannels was performed by infusing the wash solution into each microchannel. Blocking solution and wash solution for microchip were purchased from SUMITOMO BAKELITE Co. Ltd. Then a mixture of 2.0 µl of peroxidase-labeled 2nd anti-PICP antibody (Clone PC5-5, TAKARA) and 0.4 µl of sample was infused into each microchannel, followed by incubation at room temperature for 30 min. Samples were prepared according to the conventional method described above. To make a calibration curve and to examine the reproducibility in one channel or different channels, we diluted standard (640 ng/ml human PICP) with sample diluent to 600, 300, and 150 ng/ml; and these were used as samples. After washing of the microchannels with wash solution, 1.8 µl of substrate solution for the peroxidase reaction was infused into the microchannels; and the microchip was incubated for 5 min at room temperature. The integration value of luminescence intensity of each spot was measured with an ATTO Light-Capture (ATTO Corporation, Tokyo, Japan) which has a resolution of up to 62.5 µm, and analyzed by using CS Analyzer ver2.0.

### Blood sample preparation

Peripheral venous blood samples were collected from healthy subjects by standard venipuncture. All blood samples were collected into sterile centrifuge tubes, and the serum were obtained by centrifugation at 800 x g for 5 min. The serum was then stored at −80°C until used.

### Ethics

This study was approved by the Institutional Review Board on using human derivatives for biomedical research of National Institute of Advanced Industrial Science and Technology, and the Ethical Committee of University of Tokushima. All subjects provided written informed consent for the collection of samples and subsequent analysis. No current external funding sources for this study.

## Results

### Sandwich ELISA on microchip

We developed a sandwich ELISA on a COC microchip by depositing and fixing the 1st anti-PICP antibody on the surface of the microchannel with a piezoelectric inkjet printing system (Fig. 1). In the present assay, the luminescence intensity increased in a time-dependent manner after the mixture of peroxidase-labelled 2nd antibody and sample was introduced into the microchannel, and a linear relationship was observed until 30 min (data not shown). Therefore, every reaction time for 2nd antibody and antigen reaction was fixed at 30 min in the present study. The piezoelectric inkjet printing system was capable of ejecting a precise droplet volume and placing it precisely in the microchannel. Reproducibilities of the determination of the PICP concentration in one channel and different channels were examined. As shown in Table 1, slight variation of the luminescence intensity of 3 spots in 1 channel for 0, 150, 300, and 600 ng/ml PICP was observed, with the relative standard deviation (RSD) for each concentration being 4.2, 6.0, 8.1, and 7.5%, respectively. This result indicated the possibility of using a single channel with multi spotting of antibody on the microchannel for duplicate, triplicate, quadruplicate or more determinations by using only 1 channel. The RSDs of the luminescence intensity in 3 different channels for 0, 150, 300, and 600 ng/ml PICP were 4.0, 5.7, 8.1, and 8.8%, respectively (Table 2). This result indicated good reproducibility of luminescence intensity induced by the antigen-antibody reaction even in different microchannels.

**Table 1:** Reproducibility of luminescence intensity of PICP in 1 channel.

Spot number	PICP (ng/ml)			
	0	150	300	600
1	4164	19292	61840	106188
2	3932	19304	62780	102992
3	3840	17352	54020	118584
Average	3979	18649	59547	109255
RSD (%)	4.2	6.0	8.1	7.5

**Table 2:** Reproducibility of luminescence intensity of PICP in 3 different channels.

Channel number	PICP (ng/ml)			
	0	150	300	600
1	4305	16661	55275	111513
2	4125	17923	65007	128005
3	3979	18649	59547	109255
Average	4136	17744	59943	116258
RSD (%)	4.0	5.7	8.1	8.8

### Determination of serum PICP

**Figure 2:**
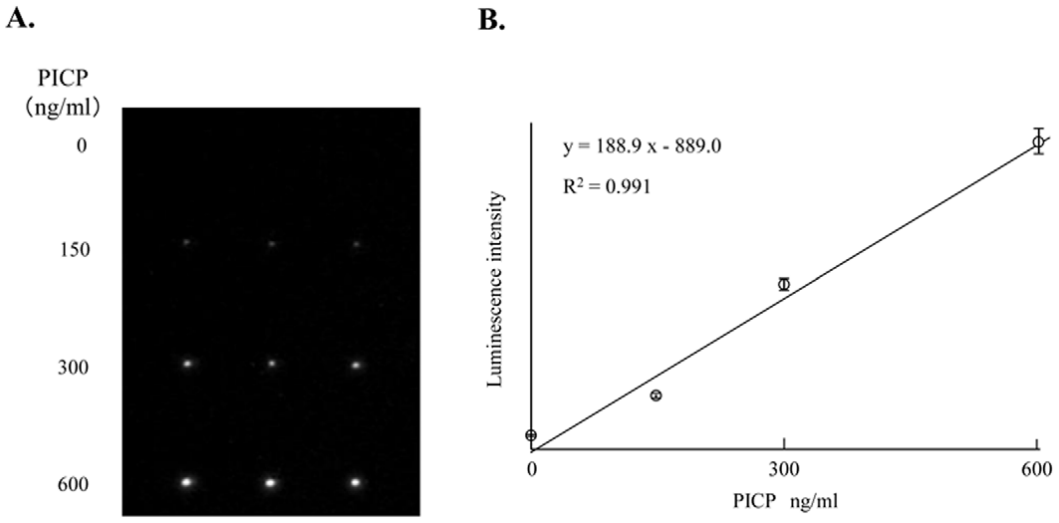
Detection of purified PICP by sandwich ELISA on a microchip. Three deposits of 1st antibody were made into each microchannel, and 0 to 600 ng/ml PICP and peroxidase-conjugated 2nd antibody solution were then infused, allowing triplicate determinations to be made in 1 channel. (A) The luminescence increased in a PICP concentration-dependent manner. (B) Standard curve for the PICP concentration *versus* luminescence intensity obtained by the assay.

**Figure 3:**
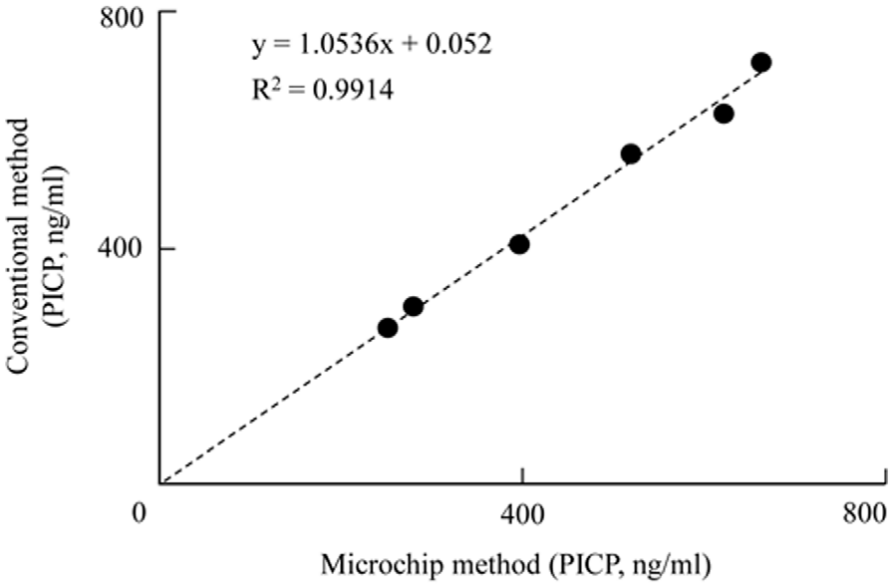
Comparative analysis of the values obtained with the microchip method and the conventional microtitration plate method. Linear regression analysis was used.

Quantitative analysis of serum PICP concentrations was performed next. The relationship between the concentrations of purified PICP in the range of 0–600 ng/ml and the luminescence intensity derived from peroxidase-conjugated 2nd anti-PICP antibody was examined (Fig. 2). Three spots of 1st anti-PICP antibody were deposited into each microchannel for triplicate determinations, and purified PICP solution (0 to 600 ng/ml) and peroxidase-labeled 2nd antibody were infused into each microchannel. The luminescence increased in a PICP concentration-dependent manner (Fig. 2A). The mean (±SD) of the luminescence intensity for the 6 different spots at each purified PICP concentration was obtained and plotted against the dose, giving a linear relationship between the range of 0–600 ng/ml PICP (R^2^ = 0.991; Fig. 2B). The detection limit was 4.7 ng/ml. This range is sufficient for clinical estimation of PICP concentrations in the blood [28], [29]. These data thus strongly indicate that this sandwich ELISA performed on a microchip with the aid of piezoelectric printing technology is suitable for the determination of blood PICP concentrations. Blood PICP concentrations in 6 subjects were then calculated by reference to the standard curve generated from data obtained from the sandwich ELISA on a microchip. Each estimated blood PICP concentration was compared with the results obtained by using the 96-well microtitration plate commonly used in clinical laboratories. As shown in Fig. 3, linear regression analysis of these PICP concentrations obtained by both methods revealed a significant relationship (R^2^ = 0.9914). This result indicated that accurate determination of blood PICP could be performed by use of our new method. Three different samples were analyzed repeatedly within 1 day or for several days for their PICP concentration (Table 3). The within-day (n = 4) reproducibility was 3.2–9.8%; and the between-days (n = 4) one, 4.4–6.8%. According to the documentation supplied in the Procollagen type I C-peptide EIA Kit, the reproducibilities of within-day and between-days were 4.5–7.4% and 4.3–6.3%, respectively, for 3 different subjects when the conventional microtitration plate was used. Thus the reproducibility of the sandwich ELISA on a microchip for the determination of the blood PICP concentration was quite comparable to that obtained with the conventional 96-well microtitration plate.

**Table 3:** Reproducibility of microchip method for the determination of PICP in serum samples.

Sample	PICP	
	Mean ± S, ng/ml	CV, %
Within day, n = 4		
1	327.4±10.3	3.2
2	479.2±47.2	9.8
3	623.7±46.4	7.4
Between days, n = 4		
4	874.5±45.0	5.2
5	580.7±39.7	6.8
6	657.9±28.9	4.4

## Discussion

To develop our quantitative sandwich ELISA using microchannels, we used an inkjet printing system, because it has been shown to deliver small reagent volumes with precise alignment and high reproducibility [27]. There are 2 main classes of inkjet printers, thermal and piezoelectric. In the thermal inkjet printers, a resistive heating element causes air bubbles to expand, expelling a liquid drop. On the other hand, voltage-induced deformation of a rectangular piezoelectric crystal squeezes ink droplets through the nozzle in the piezoelectric inkjet printer [30]. Allain et al. reported that the functionality of deposited biological materials may be compromised due to the patterning process, particularly in the case of thermal inkjet printing [31]. Several reports have shown that the functionality and stability of many biological materials may be maintained despite the high pressure involved in piezoelectric inkjet printing [25], [27], [32], [33]. Although immunoassay was performed with antibody deposition on nylon membrane by piezoelectric inkjet printing, the quantification was not shown [25]. In the present study, we employed a commercial piezoelectric inkjet printer to expel the antibody onto the microchip to avoid thermal damage to the antibody. Therefore, we could develop a sensitive and accurate sandwich ELISA on the microchip.

Although we could not compare the consumption of 1st antibody between the microchip and the microtitration plate because the 1st antibody had been precoated on the wells for the conventional method, the use of a microchip has considerable advantages; low consumption of serum (0.4 µl/microchannel versus 20 µl/well for 96-well microtitration plate) and of peroxidase-labeled 2nd antibody and substrate (2.0 µl of each/microchannel versus 100 µl/well for the plate), ease of operation, i.e., use of just 1 pipetman for infusion of each solution into the microchannel; and reduced immunoassay time (30 min for the microchip versus 3 hr for the plate). Earlier an immunosorbent assay system was integrated into a microchip, and antibody-conjugated polystyrene beads were introduced into a microchannel [19], [21]–[23]. The immunoassay time was remarkably reduced owing to the size effect of the microspace in the microchannel. For the detection of carcinoembryonic antigen in human sera, the immunoassay time was reduced to one-ninetieth of that for the conventional immunoassay with the 96-well microtitration plate [23]. This effect was obtained by an increase in the specific interface and a reduction in the diffusion distance by packaging the 41.7 µm diameter polystyrene beads in the microchannel. However, the immunoassay time in the present study was reduced to just one-sixth of that for the conventional immunoassay. This small reduction in assay time must be due to the smaller effects of specific interface and reduced diffusion distance than in the case of using beads in the microchannel. Although there is a remarkable reduction in immunoassay time when beads are used in a microchannel, the fabrication of precise dam-like structures in the microchannel is necessary to package the beads in the microspace. Precise deposition of antibody on any microchannel surface is easy with an inkjet printing system, and so there is no need for special fabrication in the microchannel. Furthermore, the plural deposition of antibodies on any part of the microchannel surface, as was shown in Fig. 2A, can be performed. So, the inkjet printer is easily applicable for multi-analysis of biomarkers by spotting different kinds of antibodies on 1 channel through the change of inkjet heads containing the antibody solution. In fact, we could detect 3 kinds of biomarkers in human blood by multi-spotting 3 kinds of antibodies on a single channel (data not shown).

In conclusion, we presented a new sandwich ELISA assay system utilizing a microchip and inkjet printer to detect serum PICP. The sandwich ELISA performed in a microchannel with deposition and fixation of the 1st antibody on the microchannel surface by use of the inkjet printing system offers considerable advantages, including low consumption of sample and reagent, reduced immunoassay time, and ease of operation. This assay on a microchip enables the determination of the blood PICP concentration with high levels of sensitivity, accuracy, and reproducibility, and is thus applicable to clinical diagnosis.
